# Modulation of Free Carbon Structures in Polysiloxane-Derived Ceramics for Anode Materials in Lithium-Ion Batteries

**DOI:** 10.3390/molecules29184461

**Published:** 2024-09-20

**Authors:** Yiling Quan, Changhao Hu, Peifeng Feng, Yujie Song, Kun Liang, Xigao Jian, Jian Xu

**Affiliations:** 1 State Key Laboratory of Fine Chemicals, Liaoning High Performance Polymer Engineering Research Center, Department of Polymer Science and Materials, School of Chemical Engineering, Dalian University of Technology, Dalian 116024, China; yilingquan@mail.dlut.edu.cn (Y.Q.);; 2 Zhejiang Key Laboratory of Data-Driven High-Safety Energy Materials and Applications, Ningbo Key Laboratory of Special Energy Materials and Chemistry, Ningbo Institute of Materials Technology and Engineering, Chinese Academy of Sciences, Ningbo 315201, China; 3 Qianwan Institute of CNITECH, Ningbo 315336, China

**Keywords:** SiOC anode, polymer-derived ceramics, free carbon, carbon nanotubes

## Abstract

Polymer-derived silicon oxycarbide (SiOC) ceramics have garnered significant attention as novel silicon-based anode materials. However, the low conductivity of SiOC ceramics is a limiting factor, reducing both their rate capability and cycling stability. Therefore, controlling the free carbon content and its degree of graphitization within SiOC is crucial for determining battery performance. In this study, we regulated the free carbon content using divinylbenzene (DVB) and controlled the graphitization of free carbon with the transition metal iron (Fe). Through a simple pyrolysis process, we synthesized SiOC ceramic materials (CF) and investigated the impact of Fe-induced changes in the carbon phase and the amorphous SiOC phase on the comprehensive electrochemical performance. The results demonstrated that increasing the DVB content in the SiOC precursor enhanced the free carbon content, while the addition of Fe promoted the graphitization of free carbon and induced the formation of carbon nanotubes (CNTs). The electrochemical performance results showed that the CF electrode material exhibited a high reversible capacity of approximately 1154.05 mAh g^−1^ at a low current density of 100 mA g^−1^ and maintained good rate capability and cycling stability after 1000 cycles at a high current density of 2000 mA g^−1^.

## 1. Introduction

In response to the swiftly escalating energy requirements for electric cars and mobile electronics, the creation of innovative lithium-ion battery electrode materials has attracted growing interest [1,2,3,4]. At the present time, commercial anode materials are predominantly carbon-based, but they fall short in matching high-performance cathode materials [5,6]. Consequently, silicon anode materials, noted for their superior theoretical capacity, are deemed the most hopeful contenders for future anode materials. However, silicon-based anode materials have significant drawbacks. Due to the lithiation/delithiation mechanism, their volume expansion during charge–discharge cycles could reach up to 300%, causing the anode material to suffer structural damage. Moreover, silicon, a semiconductor, lacks conductivity, which results in poor cycling stability [7,8,9].

Among the various silicon-based anode materials, SiOC has attracted widespread attention as an anode material that balances high capacity with cycling stability [10,11]. SiOC ceramics are composed of a chemical structure of silicon, oxygen, and carbon, featuring a microstructure made of amorphous Si-O-C tetrahedral units and disordered free carbon. The SiOC glass phase primarily contributes to a high specific capacity, while the free carbon acts as a conductive agent, providing good rate capability and cycling performance [12,13,14]. However, two key issues need to be addressed when using SiOC as an anode material: the densification and non-porous characteristics of SiOC ceramics and the inherently low electrical conductivity of SiOC ceramics, which leads to reduced initial Coulombic efficiency (ICE), cycling stability, and rate performance [15]. Common porosity-inducing methods include the sacrificial template method, where low-boiling-point polysiloxanes (PDMS) are blended with silicone oil, leaving a porous structure after the template’s removal during sintering [16]. To improve material conductivity, physical blending with carbon materials is typically employed. In general, adding highly conductive materials such as graphite [17], graphene [18], and CNTs [19] to the SiOC system is a common and effective approach to enhance electron mobility and cycling stability. However, graphene and CNTs are relatively expensive and prone to agglomeration within the system.

Therefore, some researchers chose phenyl-rich silicone oils as raw materials to increase the carbon content of ceramic materials after pyrolysis. However, due to the limited phenyl groups contained in silicone oil itself, it was not possible to control the free carbon content generated during the pyrolysis of the precursor. Consequently, researchers attempted to introduce more carbon sources and increase the free carbon content by crosslinking poly(methylhydrosiloxane) (PMHS) with divinylbenzene, thereby enhancing the carbon thermal reduction reaction at high temperatures and promoting the formation of SiC, and applied this material in the field of radar absorption. Research on carbon-rich SiOC ceramics generated at low temperatures as anode materials for lithium batteries has not been extensively reported [20,21,22]. More importantly, the free carbon phase formed in this way is a randomly distributed amorphous structure that is primarily isolated by the tetrahedral structure of the SiO_x_C_y_ glass phase. This impedes the establishment by free carbon in the ceramics of a coherent or interconnected conductive network, leading to obstructed electron transport pathways during charge–discharge processes and reduced rate performance [23,24,25]. Generally, graphitized carbon, compared to amorphous carbon, offer better structural stability and conductivity [26]. Therefore, controlling the free carbon content and its degree of graphitization within a SiOC ceramic matrix is a critical challenge. Typically, the method commonly used to enhance the graphitization of carbon is to increase the reaction temperature. However, polysiloxane-derived SiOC ceramics begin to gradually transform into crystalline SiC at temperatures above 1300 °C, losing their electrochemical activity. Therefore, to achieve the graphitization of carbon at lower temperatures, the action of catalysts (iron, ferrosilicon) is required. So, in the polysiloxane system, commonly used catalyst iron sources are FeCl_2_, iron acetate, FeAC, etc. [27,28,29]. However, the existing research work mainly reports that the introduction of transition metals, as well as the application of excess Fe or FeSi alloys for electromagnetic absorption, induces the transformation of SiOC ceramics into crystalline SiC and graphitic carbon. The application as anode materials in lithium-ion batteries and the energy storage mechanisms have not been reported in detail. Therefore, the effect of the modulation of the free carbon content and structure of polymer-converted SiOC ceramics on electrochemical performance is of a certain research value.

In this study, DVB was used as both a carbon source and a crosslinking agent, while ferric acetylacetonate (FeAC) served as a catalyst to promote the graphitization of carbon. Additional phenyl groups were introduced via hydrosilylation, and during pyrolysis, amorphous carbon was converted to graphitic carbon, and CNTs were generated under the control of metallic iron. The results showed that as the DVB content in the precursor increased, the free carbon content in the SiOC ceramic matrix also increased. Furthermore, under the catalytic effect of metallic Fe, disordered carbon was transformed into ordered carbon, leading to the internal formation of CNTs within the ceramic framework. This process enhanced the electron conveyance and the ion permeation of the anode, resulting in improved electrochemical performance.

## 2. Results and Discussion

**Figure 1:**
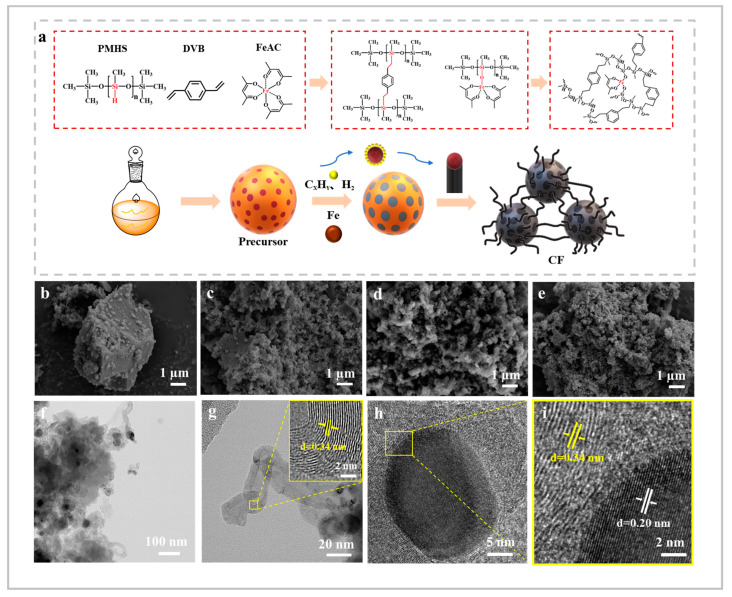
(**a**) Preparation process schematic of the CF series samples; morphological characterization of the CF series samples: (**b**) CF-25, (**c**) CF-50, (**d**) CF-75, (**e**) CF-10, (**f**–**i**) TEM and HRTEM images of CF-75.

Figure 1a provides a diagram of the preparation route for CF materials with varying DVB contents using a classical pyrolysis process catalyzed by FeAC. Figure 1b–e display the surface morphology of the CF series materials characterized by SEM. It was observed that as the DVB content increased, the ceramic matrix transitioned from a dense structure to a loose, porous structure, with more tubular structures, believed to be CNTs, gradually appearing within the ceramic matrix. Subsequently, we further observed the microscopic structure of the materials using TEM. As illustrated in Figure 1f, CF-75 contained numerous tubular structures interwoven between particles. The lattice fringes of graphitized carbon were clearly observed in the HRTEM image in Figure 1g, confirming the presence of CNTs in the material. Additionally, a small number of metal particles inside the material were observed, as shown in Figure 1h,i, which were connected to the CNTs. The lattice spacing of these particles was 0.21 nm, corresponding to the (110) plane of α-Fe. This suggests that the growth mechanism of CNTs within the material could be explained as follows: Throughout the pyrolytic conversion of the SiOC precursor, a large number of hydrocarbon and carbon–oxygen compound gas molecules serve as carbon sources, preferentially deposited on the surface of metallic iron. These molecules dissolve and polymerize on the metal surface, further growing outward in a crystalline form, ultimately forming one-dimensional CNTs [30,31,32,33].

**Figure 2:**
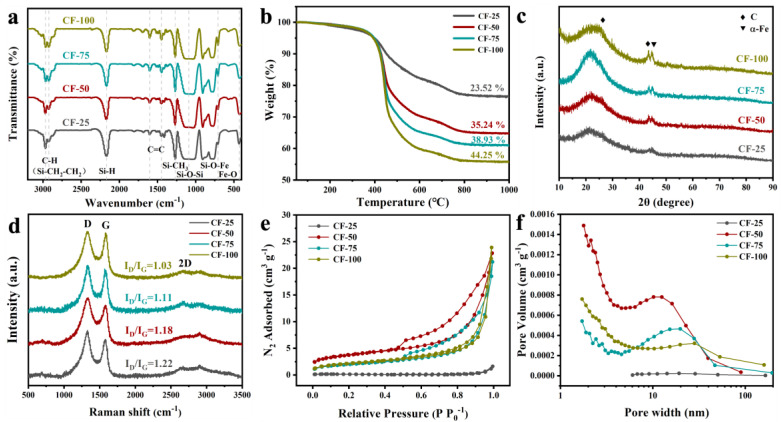
Structural characterization of the CF series samples: (**a**) FT-IR spectra, (**b**) TG spectra, (**c**) XRD spectra, (**d**) Raman spectra, (**e**) N_2_ adsorption–desorption isotherm curves, (**f**) BJH pore size distribution curves.

To study the structure of the synthesized precursors, chemical bond changes in four different precursors were analyzed using FT-IR. As illustrated in Figure 2a, all samples exhibited the Si-O-Si bond at 1200 cm^−1^, which is characteristic of the SiOC precursor backbone. With the increase in DVB content, the peaks of C=C (1600 cm^−1^ and 1450 cm^−1^) and Si-CH₂-CH₂ (2920 cm^−1^) significantly increased, while the peak intensity of Si-H (2160 cm^−1^) gradually decreased. This decrease was attributed to the silicon–hydrogen addition reaction between polymethylhydrosiloxane (PMHS) and DVB, and the increase in vinyl and phenyl content helped retain more free carbon during the pyrolysis process. Furthermore, with the introduction of FeAC, absorption peaks corresponding to C=O (1605 cm^−1^), Si-O-Fe (930 cm^−1^), and Fe-O (667 cm^−1^) appeared in the precursor. This appearance was due to the reaction of FeAC with PMHS through condensation and addition, which is consistent with literature reports on the chemical reactions forming Si-O-M (M=Fe, Co, Ni) bonds between acetylacetonate metal compounds and Si-H [34].

In order to comprehend the thermal behavior of precursors during the ceramization process, TGA was utilized to study the mass changes of iron-containing precursors with different amounts of DVB during pyrolysis, as depicted in Figure 2b. It was observed that the precursor exhibited a small thermal weight loss starting at 200 °C, which could be attributed to the decomposition of the FeAC ligands. The significant weight reduction happened in the 400–800 °C temperature span and was attributed to the breaking and rearrangement of various chemical bonds in the precursor (Si-H, Si-C, C-H) as the pyrolysis temperature increased. This was due to the emission of numerous small molecular gases, including CH_4_, CO, H_2_, and C_2_H_6_, during the ceramization process, which facilitated the conversion of organic substances into inorganic ones [35]. From the spectra, it was observed that with increasing DVB content, the mass loss of the samples showed an upward trend. This mass loss indicated the quantity of free carbon within the CF ceramics, with values of 23.52%, 35.24%, 38.93%, and 44.25%, and can be attributed to the torsion of the segments between FeAC and PMHS, which disrupted the regular crosslinking structure that PMHS and DVB should have had, thereby causing branching of the cross-linked polymer. This resulted in a reduction in the precursor’s crosslinking density and the consequent reduction in the yield of the ceramic [36]. Specifically, for CF-25, due to the lower DVB content, there was minimal steric hindrance, and considering the flexibility of the PMHS molecular chains, DVB in this sample was more likely to fully participate in crosslinking. However, as the DVB content increased, structural changes in the precursor affected the reaction. After some vinyl groups reacted with one Si-H bond, the remaining reaction sites were unable to connect with other Si-H bonds, thus not contributing to a tight crosslinking. To further verify the changes in carbon content in the pyrolysis products, the mass percentage of carbon in the CF series samples was measured using a carbon–sulfur analyzer. The carbon contents of CF-25, CF-50, CF-75, and CF-100 were 24.24%, 26.53%, 30.87%, and 34.60%, respectively. This result indicated that the free carbon content in CF ceramics could be controlled by introducing different amounts of vinyl and phenyl groups into PMHS.

To analyze the crystallization characteristics of the ceramics obtained from the precursors, XRD tests were conducted on different CF series samples following their pyrolysis at a temperature of 1000 °C. As illustrated in Figure 2c, the CF series samples primarily exhibited an amorphous structure, with a wide peak at approximately 22°, which was indicative of the SiOC glass phase. Additionally, diffraction peaks of graphite carbon were observed at 26.4° and 43.3°, corresponding to the (002) and (100) planes of graphite carbon, respectively. Notably, a diffraction peak characteristic of the (110) plane of α-Fe appeared at 44.6°, and with increasing free carbon content in the CF ceramics, the peaks of graphite carbon and α-Fe became more pronounced. This indicated that FeAC in the precursor was reduced to metallic Fe during pyrolysis, with the transition metal iron catalyzing the conversion of amorphous carbon into a structured carbon, thereby promoting the formation of graphite carbon [37]. To further compare and confirm whether it was Fe that promoted the graphitization of free carbon, we also prepared pure SiOC materials without adding FeAC and conducted XRD testing, the results of which are shown in Figure S1. As expected, the material exhibited only a broad peak, attributed to amorphous SiOC, and no other crystalline peaks.

It is well known that the extent of graphitization of carbon affects the conductivity properties of electrode materials. Therefore, the carbon structure in the CF ceramics was characterized using Raman spectroscopy, as depicted in Figure 2d. In the case of carbon-containing materials, the characteristic Raman spectrum is typically marked by two significant bands: the D band at approximately 1335 cm^−1^ and the G band at approximately 580 cm^−1^. The D band is indicative of the vibrational mode associated with disordered carbon structures, whereas the G band signifies the in-plane stretching vibration of SP^2^ hybridized carbon atoms in a more ordered arrangement [38]. Typically, the extent of carbon orderliness within a material is evaluated by examining the intensity ratio of the D peak to the G peak, represented as I_D_/I_G_. Our observations indicated that with an increase in the content of free carbon, there was a corresponding gradual rise in the G peak’s intensity. The I_D_/I_G_ values for CF-25, CF-50, CF-75, and CF-100 were 1.22, 1.80, 1.11, and 1.03, respectively. In addition, the Raman spectrum of pure SiOC with an I_D_/I_G_ value of 1.24 is provided in Figure S2. This indicated that Fe could catalyze the conversion of free carbon to graphite carbon in CF, and with increasing free carbon content, the level of induced graphitization was higher. Moreover, a 2D peak attributed to CNTs was observed around 2700 cm^−1^. This suggested that Fe catalyzed the development of CNTs from the carbon source during the pyrolysis process of the precursor, which is consistent with the TEM results.

Further, an analysis of the specific surface area and pore size distribution curves for the CF series samples was conducted, as illustrated in Figure 2e,f, and the particular data are detailed in Table S1. The CF ceramic materials demonstrated type IV isotherms accompanied by H3-type hysteresis loops, which are indicative of their mesoporous characteristics. As the free carbon content in the ceramics increased, there was an initial rise followed by a subsequent decline in the specific surface area, pore volume, and pore size of the CF materials. This trend was attributed to the introduction of FeAC, which altered the dense cross-linked structure of the precursor, while the decomposition of DVB during pyrolysis increased the release of organic small molecules. However, excessive DVB led to a reduction in specific surface area and pore volume, which might be related to the growth of CNTs in the pores. Notably, CF-75 exhibited a larger specific surface area and mesoporous structure, which makes it promising for offering increased electrochemical activity and accommodating more lithium ions.

**Figure 3:**
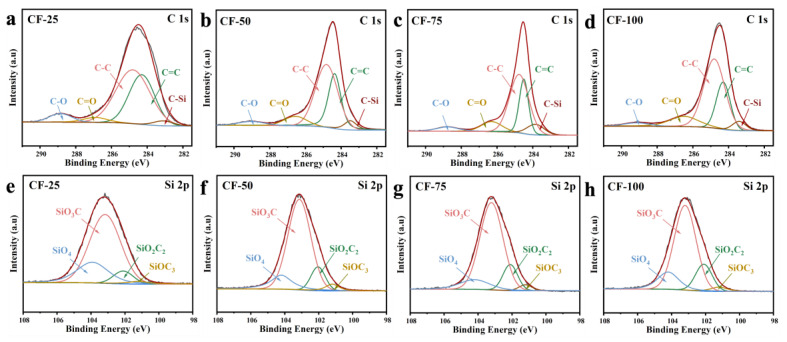
High-resolution XPS elemental spectra of C1s and Si 2p: (**a**,**e**) CF-25, (**b**,**f**) CF-50, (**c**,**g**) CF-75, and (**d**,**h**) CF-100.

The atomic structure in the CF material was further analyzed using XPS spectroscopy. Figure S3 shows the XPS full spectra of the CF ceramic materials, where characteristic peaks for C 1s, Si 2s, Si 2p, and O 1s are observable. However, Fe characteristic peaks were not identified, which might be attributed to the low 1% Fe content in the precursor and the encapsulation of metal particles by graphite carbon during CNT growth. Therefore, we focused on the electronic environment of Si 2p and C 1s. Figure 3a–d displays the C 1s fitting spectrum of the CF ceramic materials, which shows five distinct peaks corresponding to C-O, C=O, C-C, C=C, and C-Si bonds, located at 288.8 eV, 286.9 eV, 284.8 eV, 284.3 eV, and 283.1 eV, respectively. With the increase in DVB content, the proportion of C-Si bonds gradually increased, which was due to the rearrangement of more vinyl and phenyl groups with Si atoms during pyrolysis. Additionally, C=C bonds arose from graphitized carbon and CNTs. Furthermore, the composition of different structural units in the CF ceramic materials was analyzed using the Si 2p fitting spectrum shown in Figure 3e–h. Four fitted peaks were located at 104.0 eV, 103.1 eV, 102.1 eV, and 101.2 eV, corresponding to the four structural units of SiO_4_, SiO_3_C, SiO_2_C_2_, and SiOC_3_, respectively [39]. It was observed that the proportion of SiO₄ gradually decreased with increasing DVB content, while the combined proportion of SiO_3_C and SiO_2_C_2_ increased, with the CF-75 sample showing the highest proportion. This result is consistent with the C 1s fitting spectrum, and the specific proportions are listed in Table S2. Therefore, it could be confirmed that a higher DVB content promoted the formation of SiO_x_C_y_ tetrahedral units within the ceramics. However, an excessive amount of DVB led to a dominant reaction between free carbon atoms, resulting in more free carbon. Under Fe catalysis, this carbon transformed into CNTs, thereby reducing the bonding with Si.

**Figure 4:**
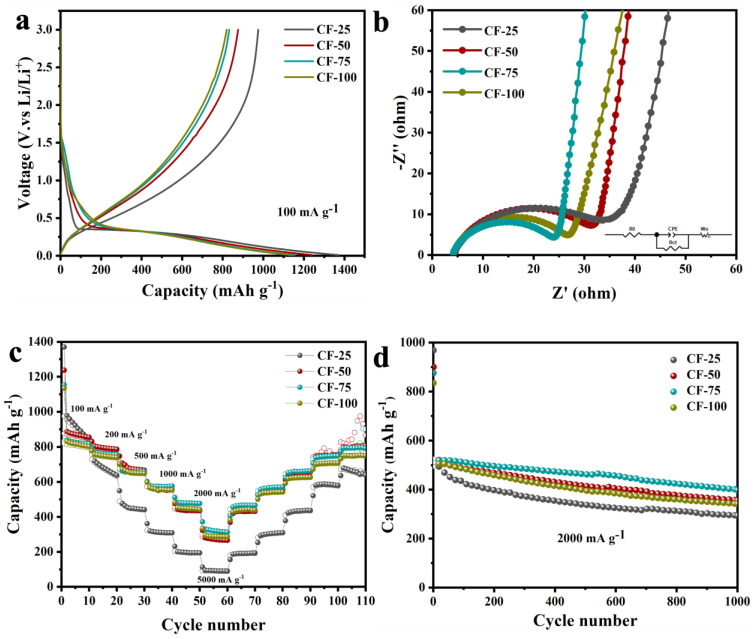
Electrochemical performance characterization of the CF series samples: (**a**) first-cycle charge–discharge curves at a current density of 100 mA g^−1^, (**b**) Nyquist plots of the CF electrodes; the inset figure is the equivalent circuit for EIS data fitting, (**c**) rate performance curves, (**d**) cycle performance curves.

The CF series electrode materials were integrated into half-cell configurations and underwent a full range of electrochemical testing. Figure 4a shows the first lap charge–discharge curves of CF-25, CF-50, CF-75, and CF-100 at a current density of 100 mA g^−1^. The first-cycle discharge capacities were 1370.35, 1237.58, 1154.05, and 1135.42 mAh g^−1^, respectively. This was directly related to the reversible lithium-ion storage phases SiO_3_C and SiO_2_C_2_ in the CF ceramics. For the CF-25 and CF-50 electrodes, the higher first-cycle capacity was attributed to the lower amount of free carbon and the relatively higher presence of partially reversible SiO_4_ phases. The ICE served as a crucial metric for evaluating the efficiency of battery capacity utilization. Figure S4 shows the charge–discharge curves of CF-25, CF-50, CF-75, and CF-100 electrode materials over the first three cycles at a current density of 100 mA g^−1^. The ICE values were 71.16%, 70.77%, 72.10%, and 72.18%, respectively, significantly higher than the ICE value of pure SiOC ceramic anode materials (60%) [40]. Generally, the first-cycle capacity loss is attributed to the formation of a SEI layer and to irreversible lithiation reactions, such as the formation of Li_4_SiO_4_ and Li_2_O. Therefore, the CF-75 electrode, which had a lower amount of irreversible lithiation phases (SiOC_3_ and SiO_4_) and a higher carbon content, demonstrated better lithiation performance. From the charge–discharge curves of the CF-75 electrode material in Figure S5, it is evident that with increasing cycle number, the Coulombic efficiency gradually increased. The nearly overlapping curves starting from the second cycle also indicate the superior cycle stability of the electrode material. In addition, electrochemical impedance (EIS) tests were performed on the CF material in order to determine the effect of structural changes on conductivity. As shown in Figure 4b, the Nyquist diagram of the CF electrode consists of a semicircle in the high-frequency region and a slash line in the low-frequency region, which represent the charge transfer resistance (Rct) and the Warburg impedance (Wo), respectively, of Li^+^ in the electrode material. The Rct values of 28.13 Ω, 24.83 Ω, 18.52 Ω, and 20.36 Ω for CF-25, CF-50, CF-75, and CF-100 were obtained from the equivalent circuit fitting results, which indicated that CF-75 had the lowest charge transfer resistance and the highest conductivity. Meanwhile, CF-75 had the highest slope in the low-frequency region, indicating the lowest diffusion impedance. Therefore, with the increase in free carbon content in SiOC ceramics and Fe-catalyzed graphitization of carbon and the formation of carbon nanotubes, the enhancement of electron transport and the diffusion of Li^+^ were facilitated.

In order to assess the potential application of the electrode materials, rate performance tests were conducted on the four CF electrode materials at different current densities, as shown in Figure 4c. CF-25 exhibited a significant decrease in capacity with increasing current density, indicating that the dense structure and low conductivity of the low-carbon CF ceramics hindered the rapid intercalation and deintercalation of lithium ions. In contrast, CF-75, with its higher graphite carbon content, achieved a capacity of 875.95 mAh g^−1^ at a current density of 100 mA g^−1^. This suggests that the higher content of reversible lithium storage phases SiO_3_C and SiO_2_C_2_, along with the interconnected CNTs and porous structure in the ceramics, enhanced both ion diffusion capabilities and electron transport. The cycle stability of the electrode materials was also a critical indicator for evaluating the electrochemical performance. Figure 4d shows the cycle stability curves of the four CF electrode materials after 1000 cycles of charge and discharge at 2000 mA g^−1^. CF-50, CF-75, and CF-100 exhibited similar long-cycle performance, with high capacities and excellent cycle stability. Among them, CF-75 retained the highest capacity of 401.40 mAh g^−1^ after 1000 cycles. This suggests that a higher content of graphitic carbon and CNTs in the ceramics suppressed the volume expansion of the alloy phase during lithium insertion and delithiation, thereby improving the cycling stability. From these results, it could be concluded that the precursor structure design achieved the intended effect. The synergistic effect of graphite carbon, CNTs, and porosity in the prepared composite materials significantly stabilized the structural integrity and addressed the issues of inadequate rate performance and cycle stability in the presence of high current densities, typical in SiOC materials.

## 3. Materials and Methods

### 3.1. Materials

Polymethylhydrogensiloxane (PMHS, MW = 1900, Macklin), iron acetylacetonate (Fe(acac)₃, 98%, Aladdin), divinylbenzene (DVB, technical grade, 80%, Aladdin), Karstedt’s catalyst (2% of Pt element in xylene, Aladdin), and toluene (AR, Aladdin) were used as received.

### 3.2. Preparation of the CF Materials

A mixture was prepared by thoroughly combining 2.1 g of PMHS, 1 μL of Karstedt catalyst, 6 g of toluene, and iron acetylacetonate (with iron content constituting 1% of the matrix weight). DVB was then added to the mixture in varying proportions (25%, 50%, 75%, and 100% of the PMHS weight), which was followed by stirring. The mixture was subjected to magnetic stirring at 80 °C for 3 h, which was subsequently followed by a 4 h process in a vacuum oven set at 110 °C, during which a cold trap and a vacuum pump were connected to remove the solvent. The temperature was then increased to 140 °C, and the mixture was further heated for 4 h to complete the curing process, which resulted in the formation of the precursor. The precursor was then thoroughly ground and positioned inside a tubular furnace. It was warmed to 700 °C at a speed of 5 °C/min under an argon environment and held at that temperature for 2 h. Subsequently, the temperature was further raised to 1000 °C at a speed of 4 °C/min, and this condition was maintained for 2 h. The final product was thoroughly ground and designated as CF-25, CF-50, CF-75, and CF-100. SiOC (DVB accounting for 75% of the mass of the PMHS.) was synthesized using no FeAC addition and the same method as described above.

### 3.3. Material Characterization

The microscopic morphology and lattice spacing of the materials were observed using a scanning electron microscope (SEM, G300, Zeiss, Jena, Germany) and a transmission electron microscope (TEM, Talos F200X, Thermo Scientific, Waltham, MA, USA). With the aid of a Fourier transform infrared spectroscopy apparatus (INVENIO R, Bruker, Borken, Germany), the CF precursor’s chemical bonding structure was confirmed. Powder X-ray diffraction (XRD, ADVANCE D8, Bruker, Karlsruhe, Germany) was used to collect the crystal structure of the materials. The surface chemical composition and bonding structure of the CF materials were analyzed by X-ray photoelectron spectroscopy (XPS, AXIS-SUPRA+, Kyoto, Japan). Thermogravimetric analysis (TG, 209 F1 Libra, Netzsch, Selb, Germany) was employed to characterize the chemical bonds in the precursors. Information regarding the carbon content in the CF ceramic samples was acquired through the use of a carbon–sulfur analyzer (CS844, St. Joseph, MI, USA). A laser confocal Raman spectrometer (LabRAM HR Evolution, Horiba, Kyoto, Japan), was deployed to determine the extent of carbon graphitization in the CF materials. Additionally, the specific surface area and pore size distribution were measured with a physical adsorption analyzer (ASAP2460, Micromeritics, Norcross, GA, USA).

### 3.4. Electrochemical Measurements

For the assembly of operational electrodes, a mixture was created by blending the active substance with conductive carbon (Super P) and polyvinylidene fluoride (PVDF) in a weight ratio of 8:1:1, using N-methyl-2-pyrrolidone (NMP) as the solvent to produce a slurry. This slurry was uniformly spread onto copper foil substrates and allowed to dry in a vacuum oven at a temperature of 80 °C for a full night. The coated copper foil was trimmed into discs of 14 mm diameter, with each disc containing approximately 0.8–1.2 mg of active material. Finally, coin cells with designation 2032 (CR2032) were constructed within an argon-enriched glove box, employing lithium metal to serve as the counter electrode. A glass fiber membrane from Whatman, model GF/D, was utilized as the separator. The electrolyte was a solution containing 1 M LiPF_6_ in a blend of ethylene carbonate, diethyl carbonate, and a small amount of fluoroethylene carbonate, mixed in a volume ratio of 1:1:0.05. The electrochemical performance of the cells was evaluated through continuous-current charge–discharge cycles, conducted within a voltage window spanning from 0.01 V to 3 V. Also, Electrochemical impedance spectroscopy (EIS) was also performed on an electrochemical workstation (VMP-3e, French) in a frequency range from 0.1 Hz to 100 kHz.

## 4. Conclusions

In summary, CF composites were successfully prepared by regulating the content of free carbon in CF ceramics using DVB and employing Fe, derived from the decomposition of FeAC, as a catalyst to promote the graphitization of free carbon and the growth of CNTs. The results demonstrated that this anode material maintained structural integrity throughout the charge–discharge cycles, exhibiting remarkable capabilities in rate performance and cycle life. Notably, after 1000 cycles at a high current density of 2000 mA g^−1^, a specific capacity of 401.40 mAh g^−1^ was retained. In conclusion, this research provides a simple and effective solution for controlling the amount, degree of graphitization, and morphology of free carbon in SiOC ceramics, offering valuable insights for the utilization of these materials in rapid charging and discharging and suggesting their excellent cycling capability in lithium-ion batteries.

## Data Availability

The data presented in this study are available upon request from the corresponding author.

## Supplementary Materials

The following supporting information can be downloaded at https://www.mdpi.com/article/10.3390/molecules29184461/s1, Figure S1: XRD spectrum of a SiOC sample; Figure S2: Raman spectrum of a SiOC sample; Figure S3: XPS full spectrum of the CF series samples; Figure S4 (a–d): Charge–discharge profiles of the CF series samples for the first three revolutions at 100 mA g^−1^ current density; Figure S5: Charge–discharge curves of CF-75 at a current density of 100 mA g^−1^; Table S1: Specific surface area and pore volume and pore size of the CF series samples; Table S2: Si 2p XPS spectroscopic results for the CF series samples.
