# Supplementary material for: Modulation of Free Carbon Structures in Polysiloxane-Derived Ceramics for Anode Materials in Lithium-Ion Batteries
Source: Molecules. 2024 Sep 20;29(18):4461. doi: 10.3390/molecules29184461 (PMC11434428; doi:10.3390/molecules29184461)
Supplement: Supplementary file 1 [file molecules-29-04461-s001.zip › molecules-3203215-supplementary.pdf]

Supplementary Material

Modulation of Free Carbon Structures in Polysiloxane-Derived Ceramics for Anode Materials in Lithium-Ion Batteries

Yiling Quan [a,b,c], Changhao Hu [a,b,c], Peifeng Feng [a,b,c], Yujie Song [b,c *], Kun Liang [b,c *], Xigao Jian [a], Jian Xu [a,b,c*]

[a]*State Key Laboratory of Fine Chemicals, Liaoning High Performance Polymer Engineering Research Center, Department of Polymer Science and Materials, School of Chemical Engineering, Dalian University of Technology, Dalian 116024, China; yilingquan@mail.dlut.edu.cn (Y.Q.); huchanghao666@163.com (C.H.); fpf999@mail.dlut.edu.cn (P.F.); jian4616@dlut.edu.cn (X.J.)*

[b]*Zhejiang Key Laboratory of Data-Driven High-Safety Energy Materials and Applications, Ningbo Key Laboratory of Special Energy Materials and Chemistry, Ningbo Institute of Materials Technology and Engineering, Chinese Academy of Sciences, Ningbo 315201, China.*

[c]*Qianwan Institute of CNITECH, Ningbo 315336, China*

** Corresponding author: songyujie@nimte.ac.cn (Y.S.); kliang@nimte.ac.cn (K.L.); xujian1028@dlut.edu.cn (J.X.)*

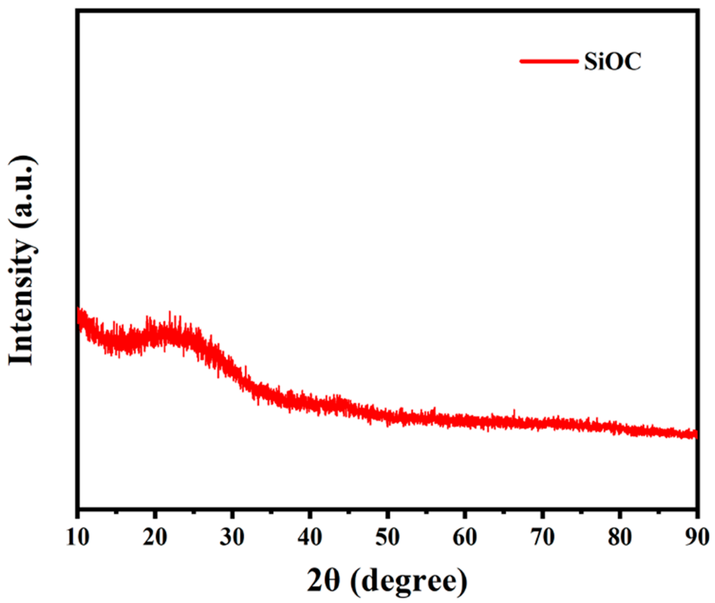

Figure S1 XRD spectrum of SiOC sample.

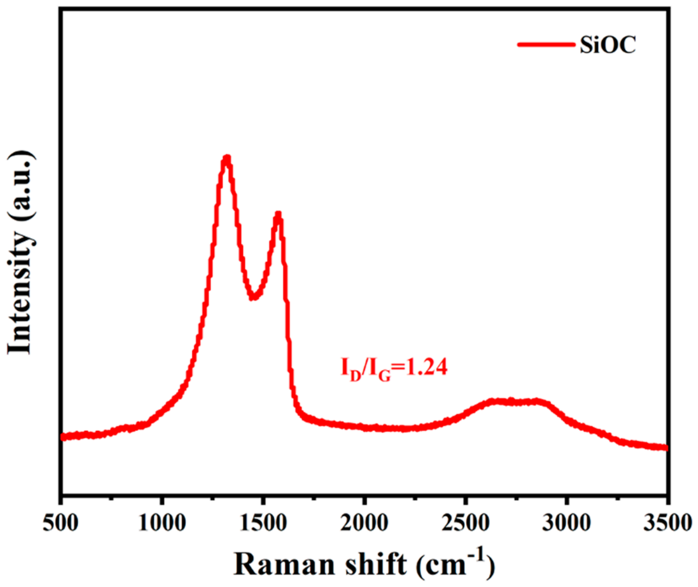

Figure S2 Raman spectrum of SiOC sample.

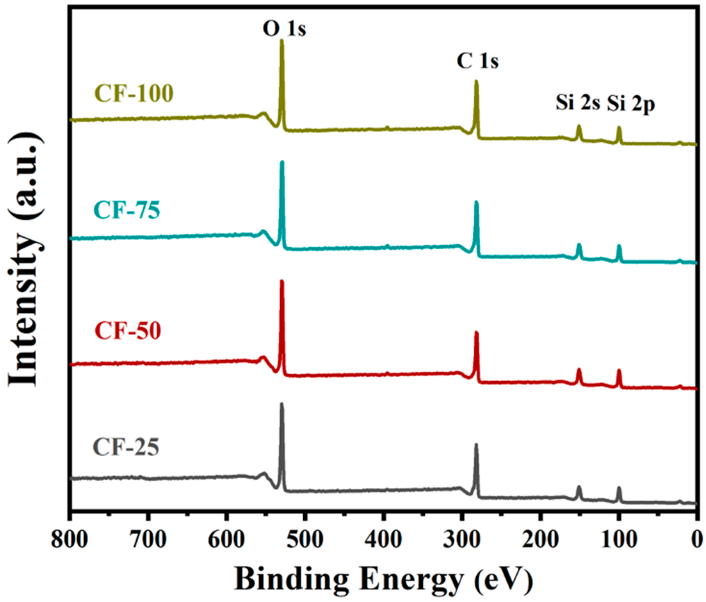

Figure S3 XPS full spectrum of CF series samples.

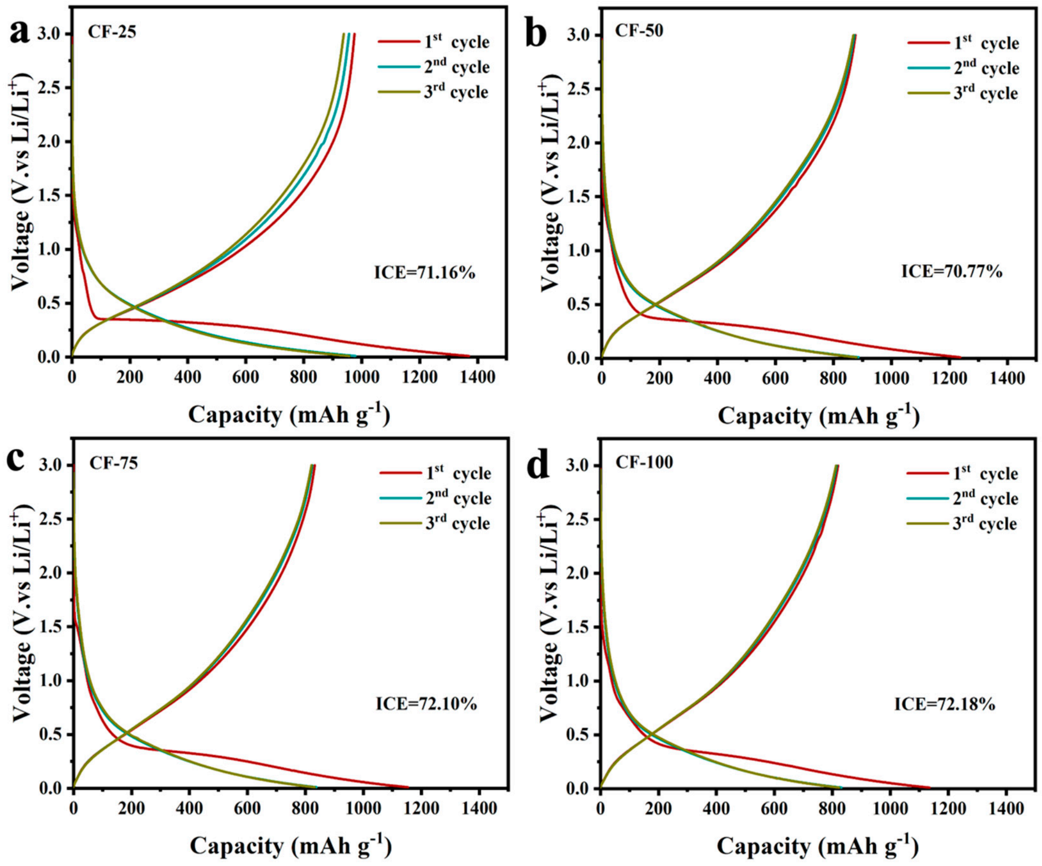

Figure S4 (a-d) Charge-discharge profiles of CF series samples for the first three revolutions at 100 mA g^{-1} current density.

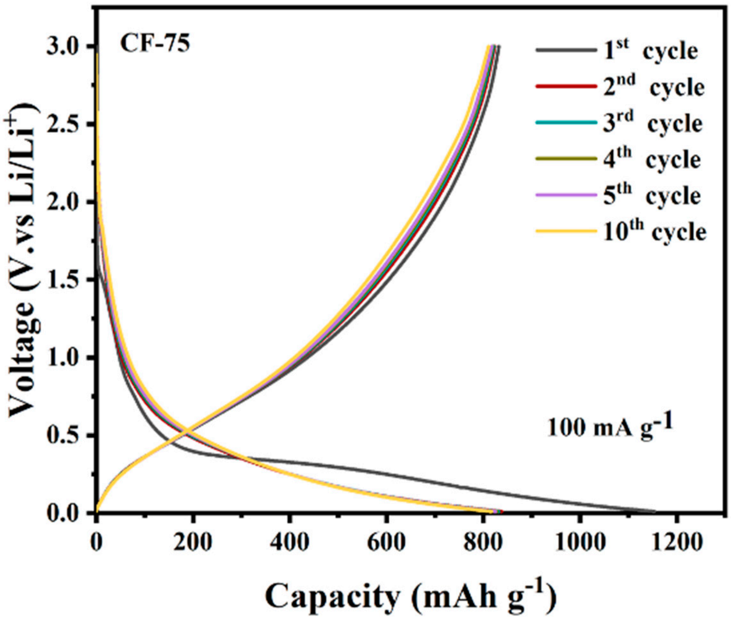

Figure S5 Charge-discharge curves of CF-75 at a current density of 100 mA g^{-1}

Table S1 Specific surface area and pore volume and pore size of CF series samples.

Sample	Specific surface area (m^2 g^{-1})	Pore volume (cm^3 g^{-1})	Pore diameter (nm)
CF-25	0.344	0.0025	37.77
CF-50	12.81	0.0353	13.01
CF-75	7.11	0.0321	21.73
CF-100	7.63	0.0363	22.21

Table S2 Si 2p XPS spectroscopic results of CF series samples.

Sample	The ratio of the peak integral area (%)			
	SiO_4	SiO_3C	SiO_2C_2	$SiOC_3$
CF-25	24.07	65.62	7.74	2.58
CF-50	14.27	70.22	11.50	4.01
CF-75	11.84	71.83	13.51	2.82
CF-100	15.60	65.54	15.74	3.11
